# Supplementary material for: The Oncosuppressive Properties of KCTD1: Its Role in Cell Growth and Mobility
Source: Biology (Basel). 2023 Mar 21;12(3):481. doi: 10.3390/biology12030481 (PMC10045846; doi:10.3390/biology12030481)

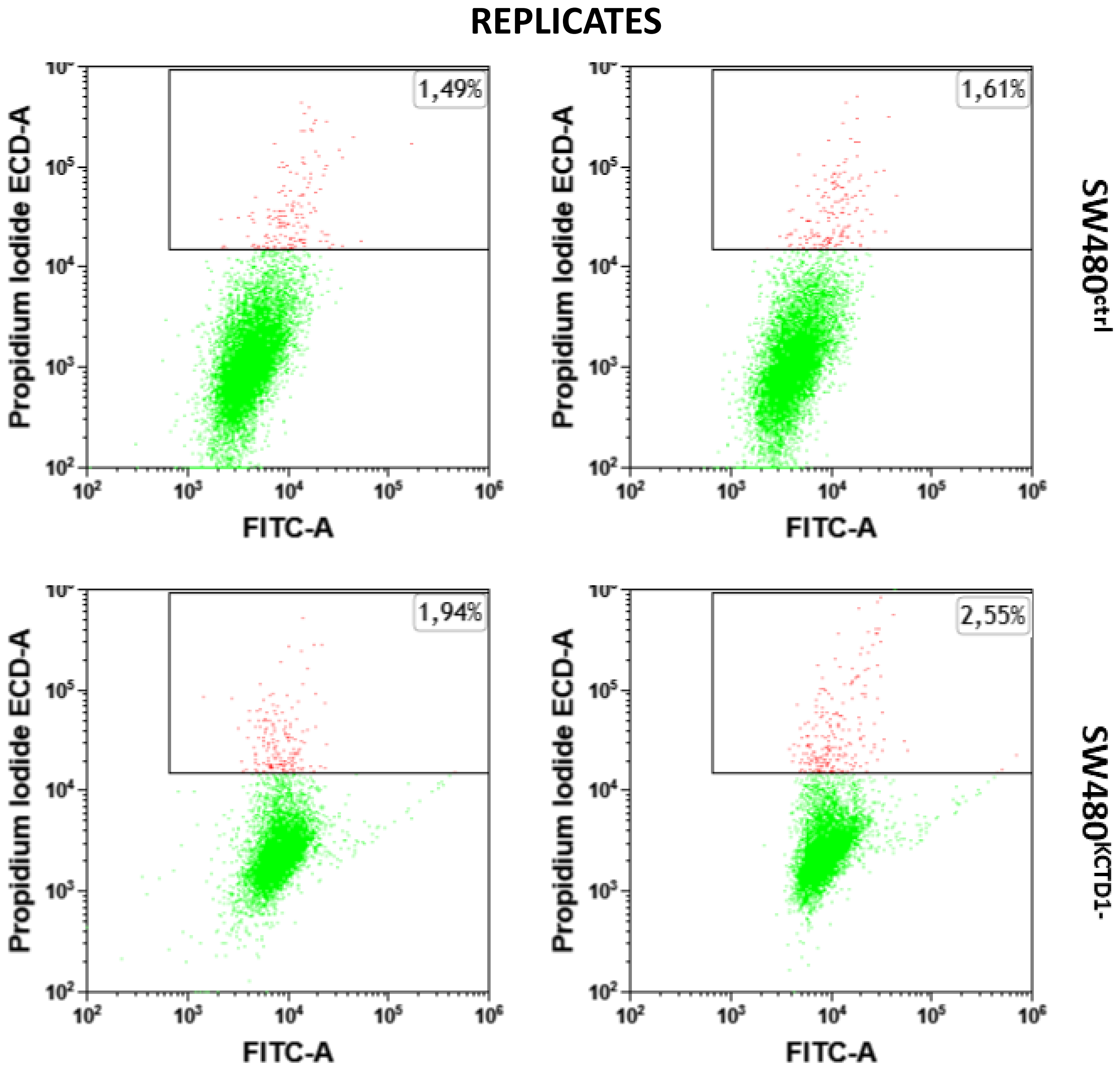

Supplementary Figure S1. Replicates of FACS analysis of the Figure 1B. Propidium Iodide VS FITC Dot plots analysis of SW480^{ctrl} and SW480^{KCTD1-} cells. Numbers report the Propidium Iodide positive cells.

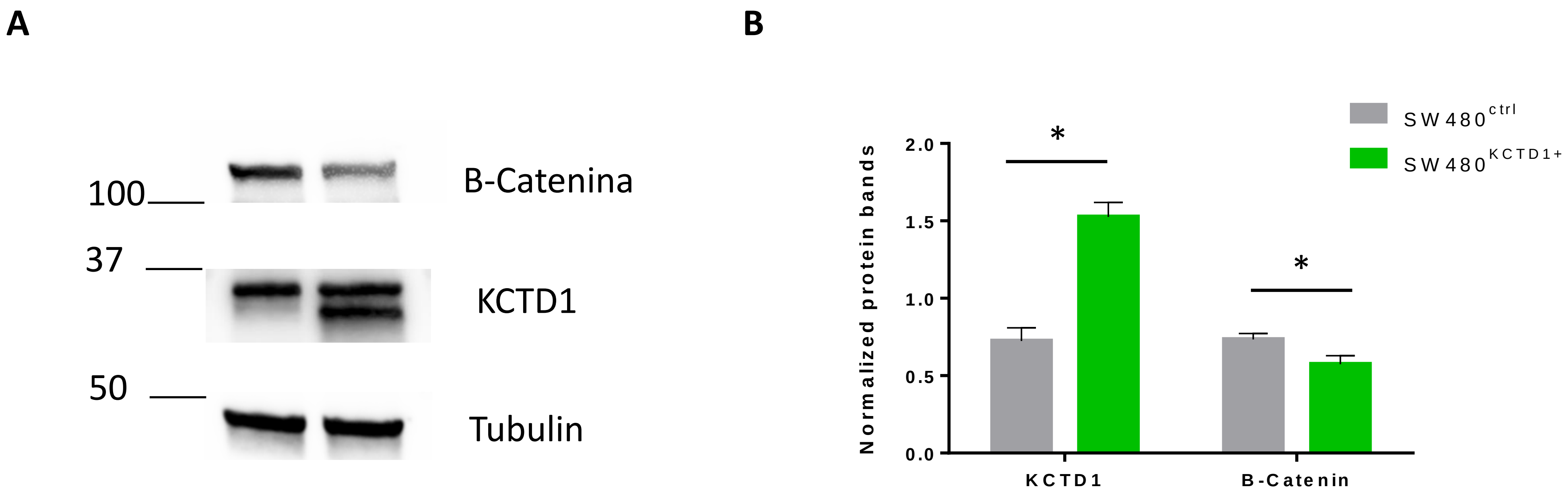

Supplementary Figure S2. Western blot analyses of the indicated protein (A) and protein band quantifications (B) were performed on SW480^{ctrl} (grey bars) and SW480^{KCTD1+} (green bars) after 24 h of over-expression. Numbers represent molecular weight of proteins expressed in kDa. ns = not significant; * = p-value < 0.05.

	SW480^{ctrl}	SW480^{KCTD1-}
Standard Deviation	359711,83	843077,83

	SW480^{ctrl}	SW480^{KCTD1-}
Standard Deviation	11284,27	37780,74

	SW480^{ctrl}	SW480^{KCTD1-}
Standard Deviation	132369,16	129130,45

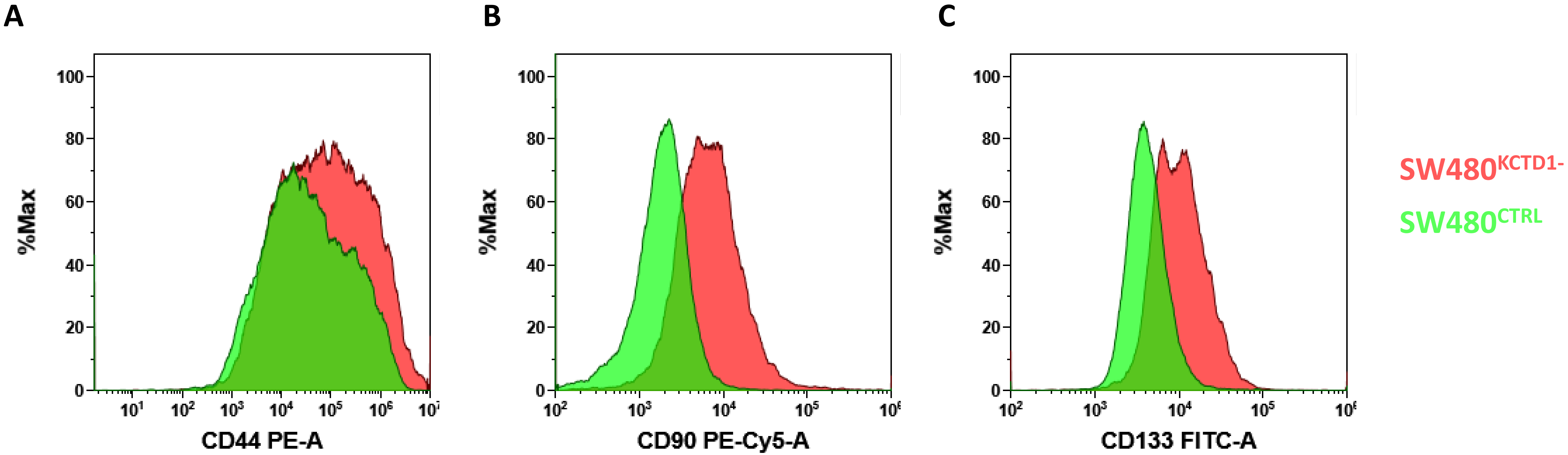

Supplementary Figure S3. Cytofluorimetric analysis of CD44 (A), CD90 (B) and CD133 (C) membrane expression in SW480^{KCTD1-} and SW480^{ctrl} cells

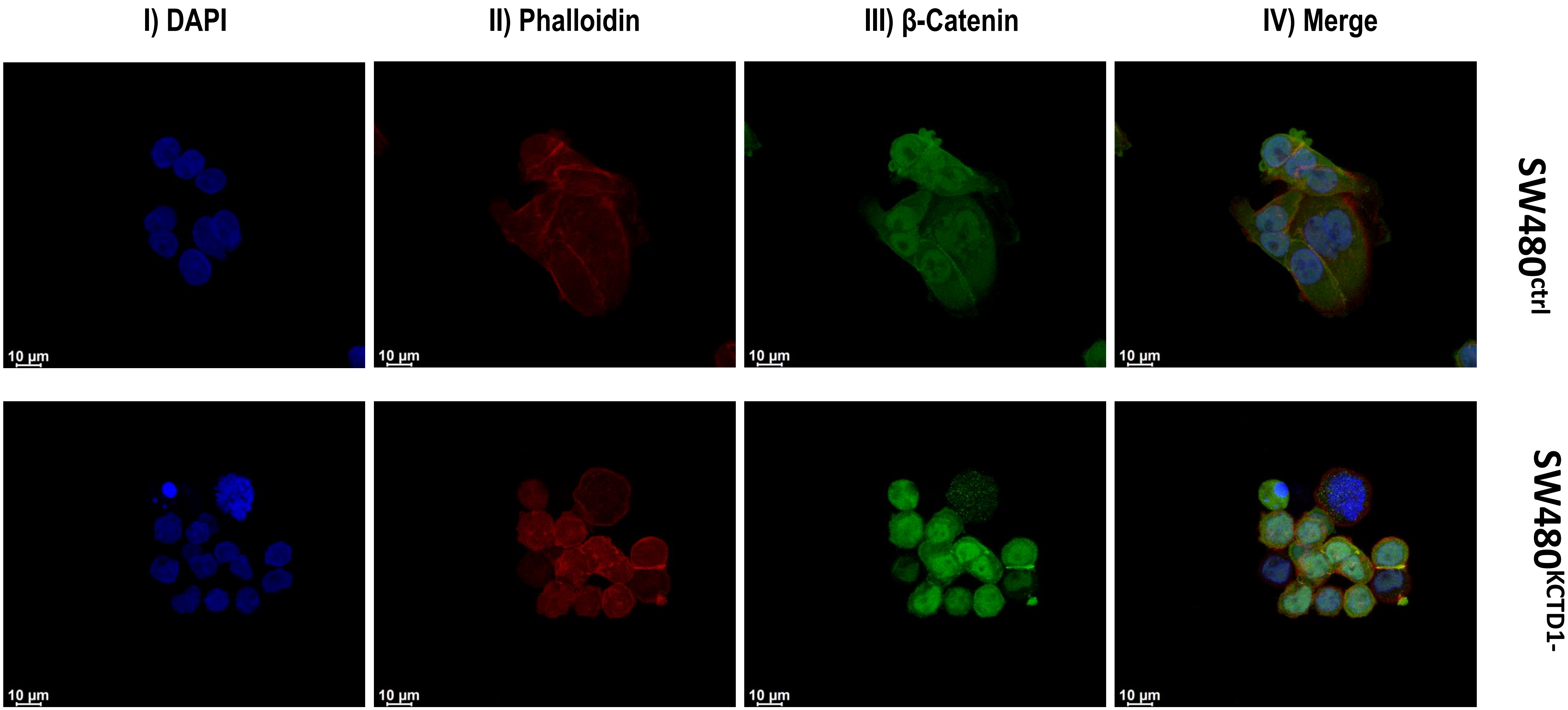

Supplementary Figure S4. Immunofluorescence analyses of SW480ctrl (upper panels) and SW480KCTD1- (lower panels). I) Nuclei staining with DAPI (blue). II) Phalloidin-Dye light 554. III) β-catenin staining with Alexa588-conjugated secondary antibody. IV) Merge of DAPI, Dye light 554 and Alexa 488. Magnification 63 ×. Scale bars 10 μm.

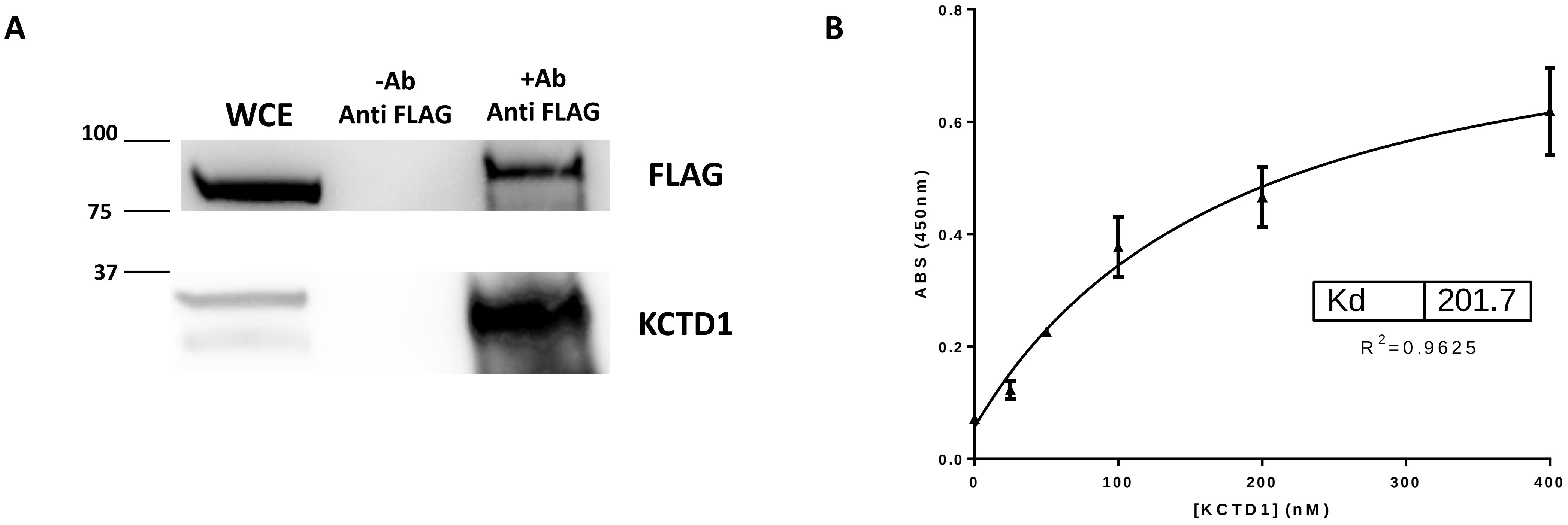

Supplementary Figure S5. A) SW480 over-expressing FLAG-IKK-β and KCTD1 cell extracts were incubated with or without anti-FLAG antibody, precipitated by Protein A/G beads, and detected by Western blots using the reported antibody. (+ Ab Anti FLAG) = elution step in the presence of Anti Flag antibody . (− Ab Anti FLAG) = elution without antibody. WCE whole cell extract. The numbers represent the molecular weight of the protein marker expressed in kDa. B) Binding of recombinant KCTD1 with IKK-B kinase by ELISA. Different KCTD1 concentration are tested (from 400nM to 25 nM). Kd was calculated using One-site Total equation.

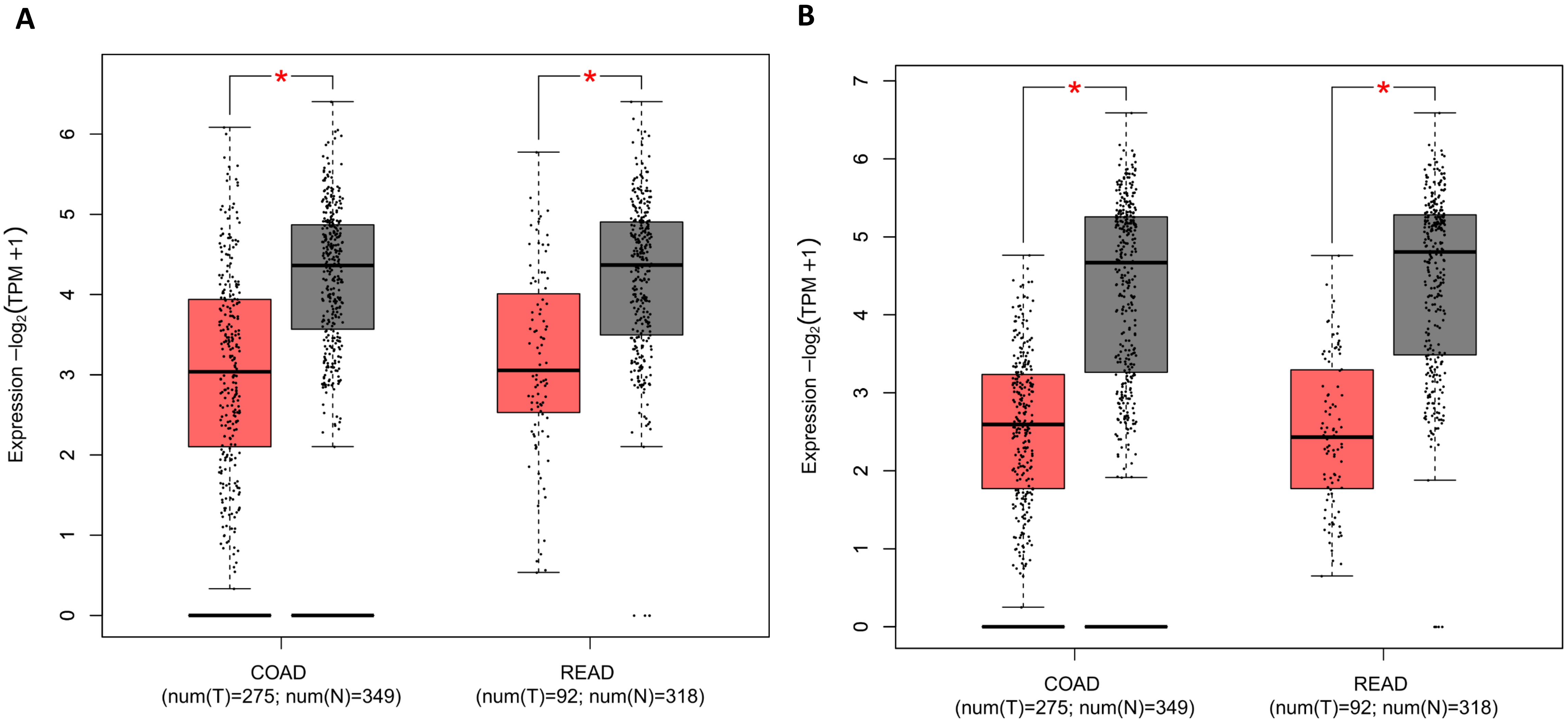

Supplementary Figure S6. KCTD12 (A) and KCTD15 (B) mRNA expression in Colon Adenocarcinoma (COAD) and Rectum Adenocarcinoma (READ) and normal tissues (GEPIA). Box plots show median mRNA expression of KCTDs in tumor tissues (red plots) and the corresponding normal tissues (gray plots). Axis units are Log2 (TPM + 1). * red = p-value < 0.01. T = tumor tissues. N = normal tissues.

Supplementary Figure S7:
Uncropped Western Blot Images

Figure 1A’s uncropped Western blot image

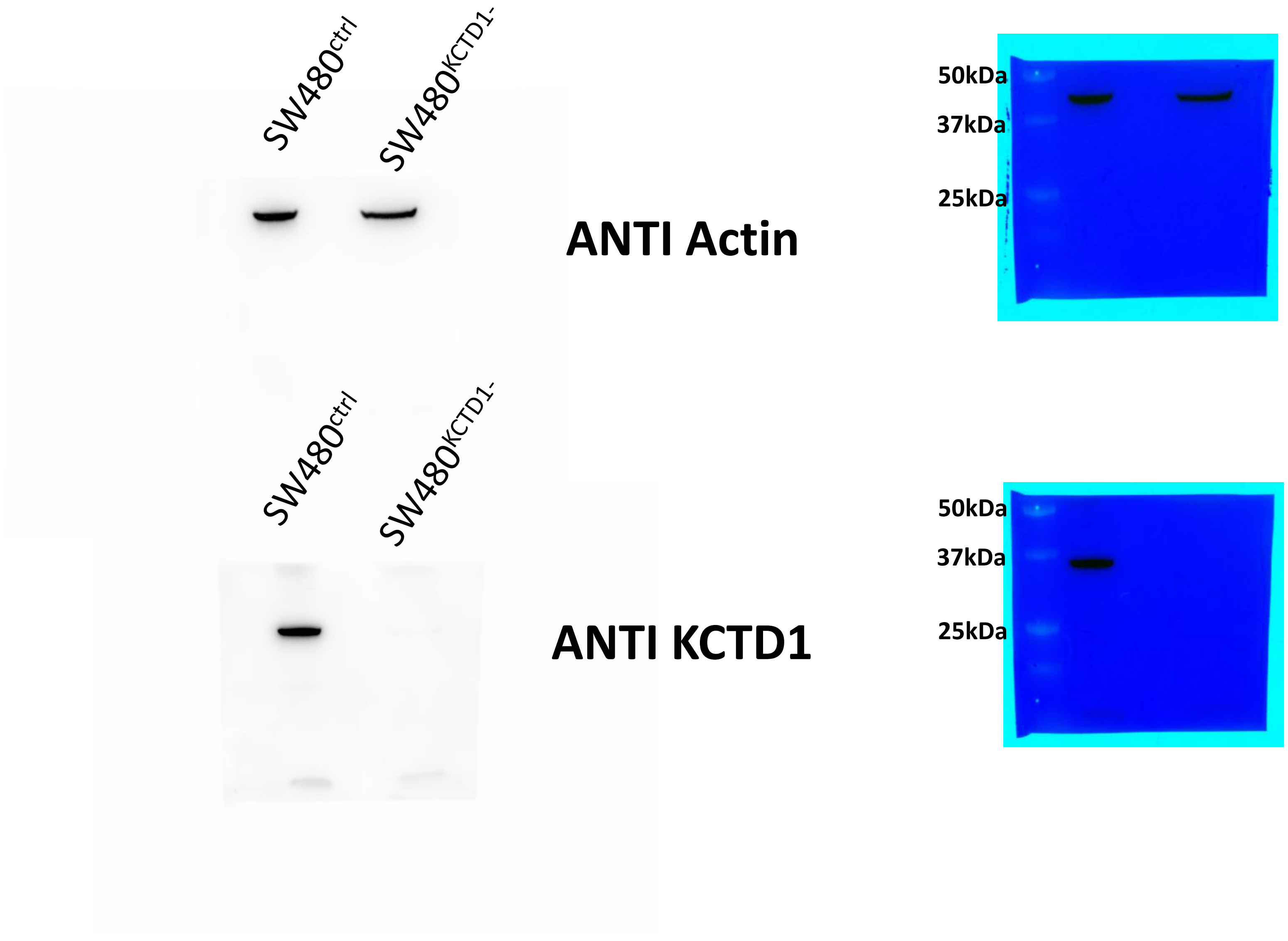

Figure 1D’s uncropped Western blot image

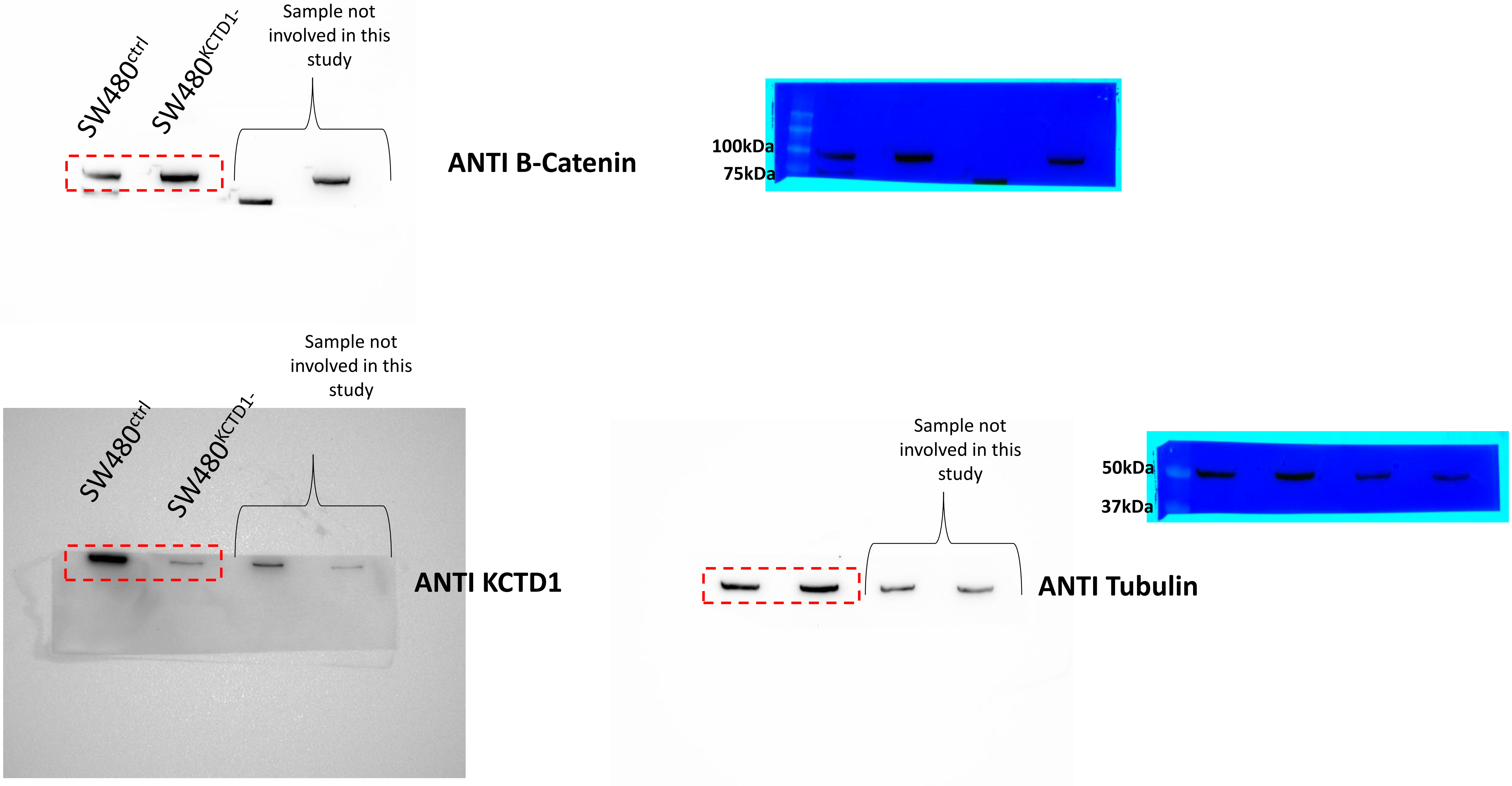

Figure 5C's uncropped Western blot image

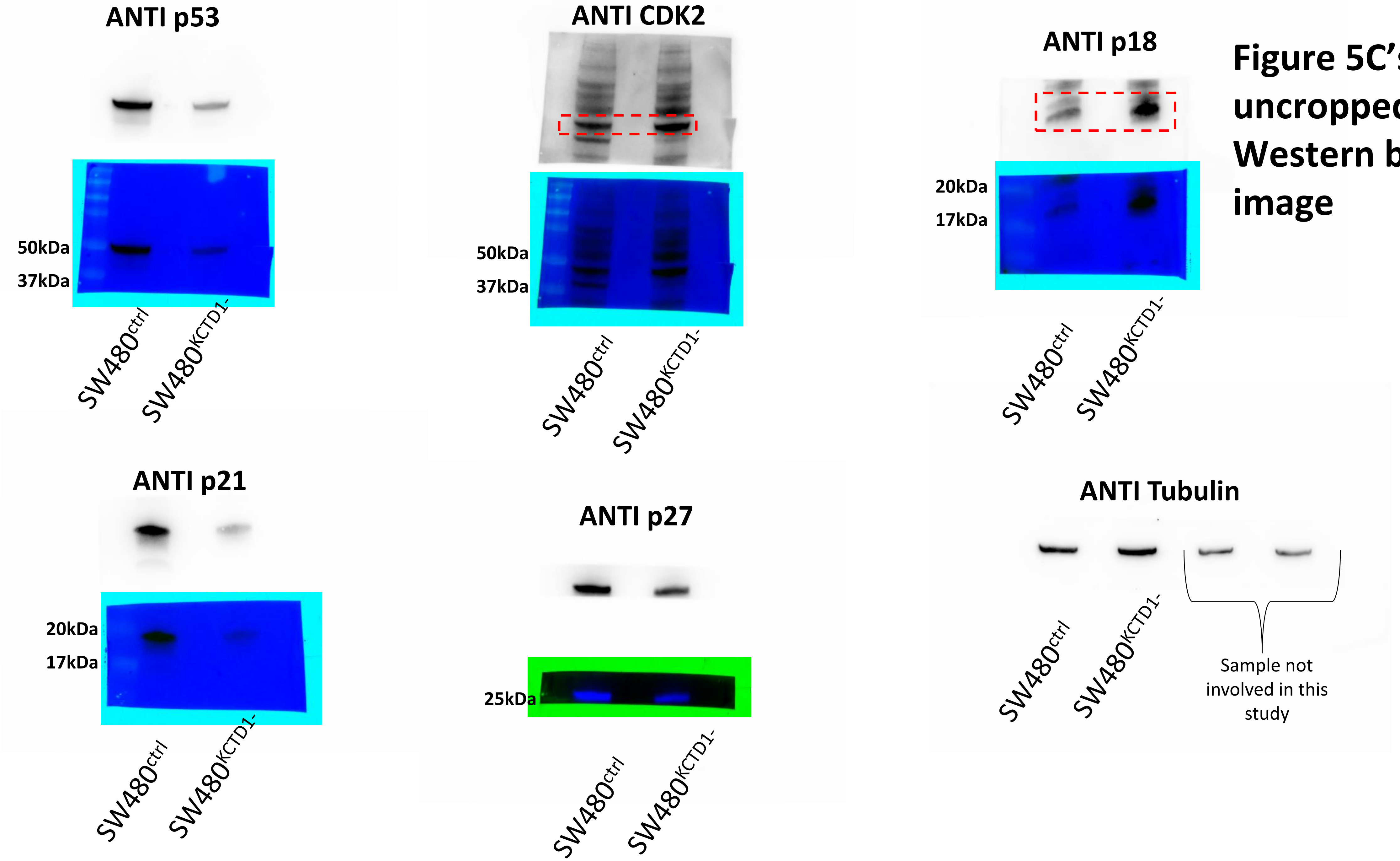

Figure 7A’s uncropped Western blot image

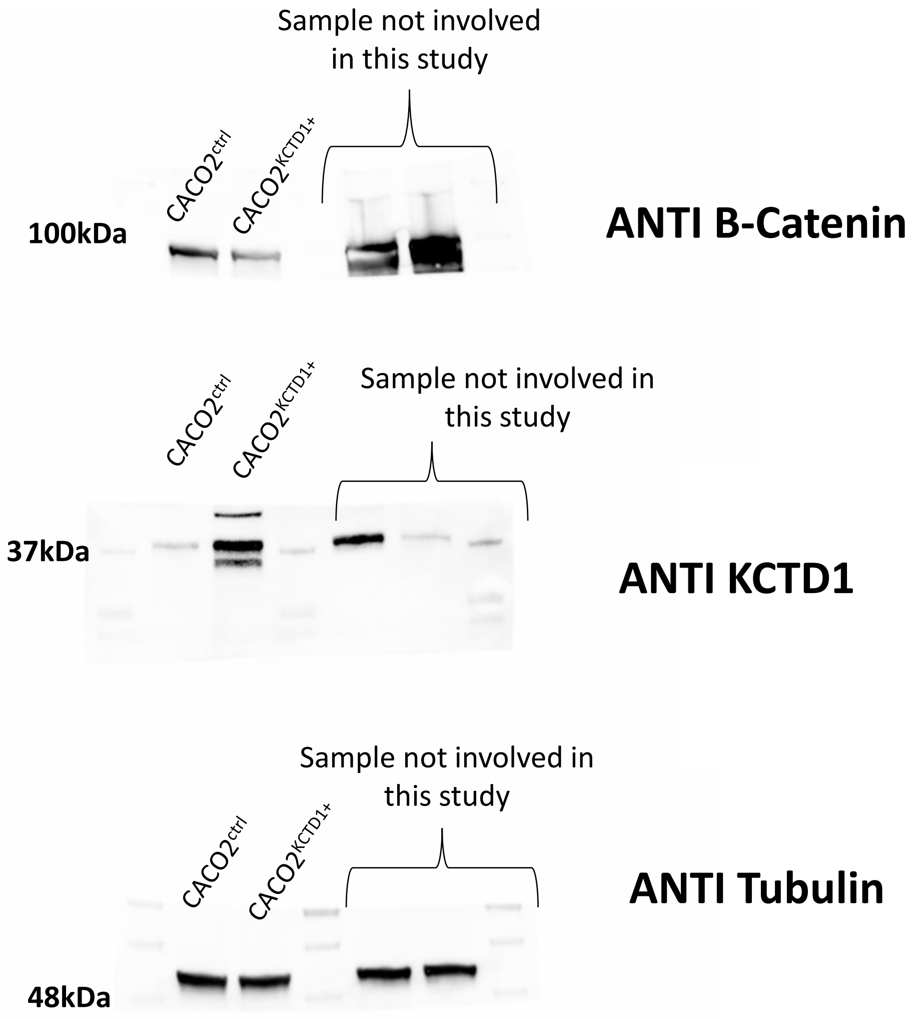

Supplementary Figure S5A's uncropped Western blot image

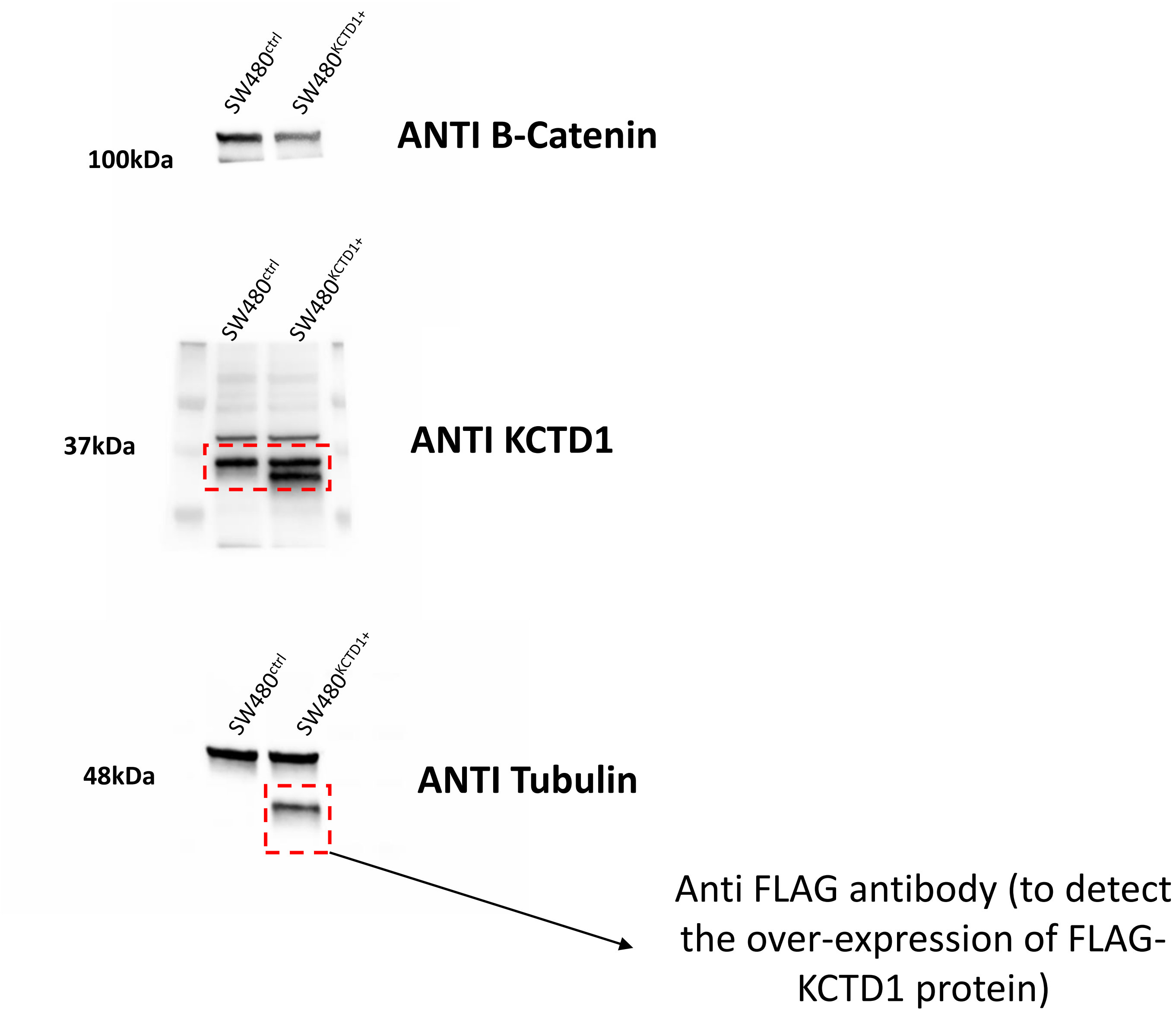

Supplement: Supplementary file 1 [file biology-12-00481-s001.zip › biology-2213501-supplementary.pdf]
